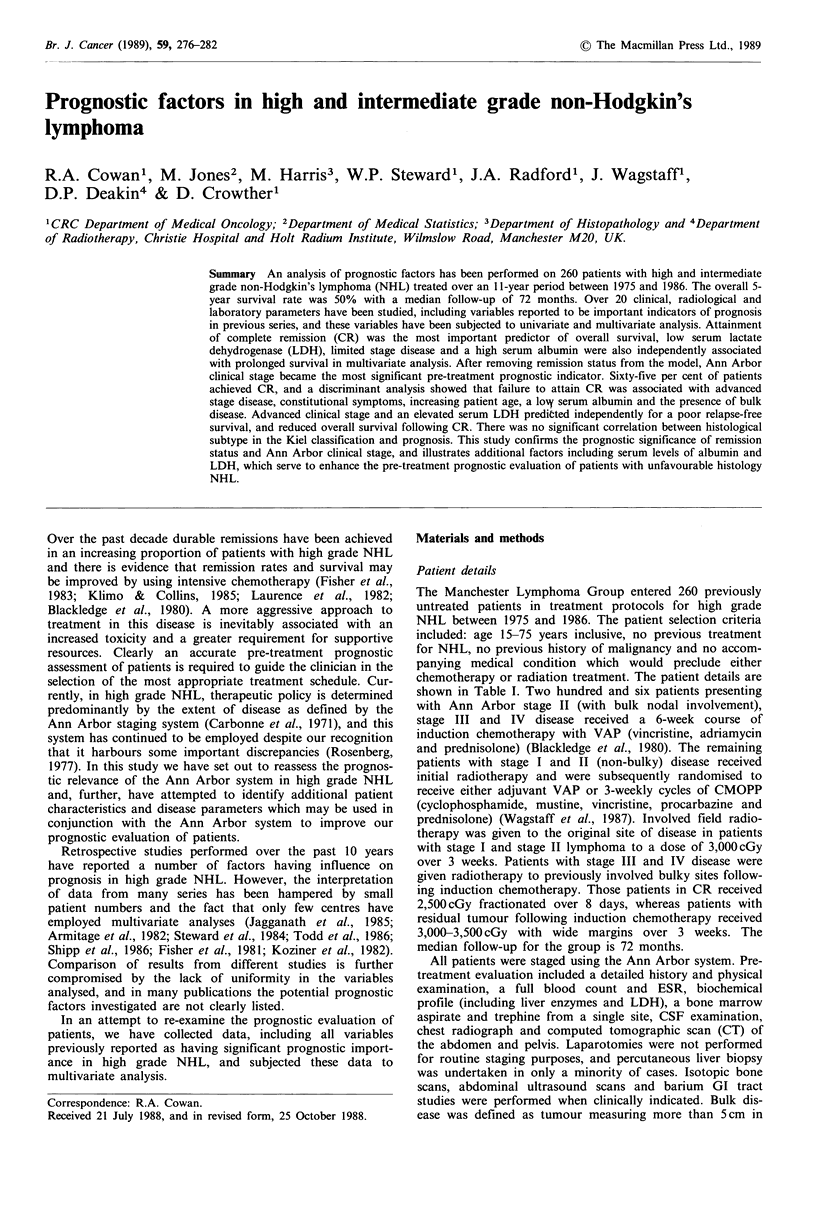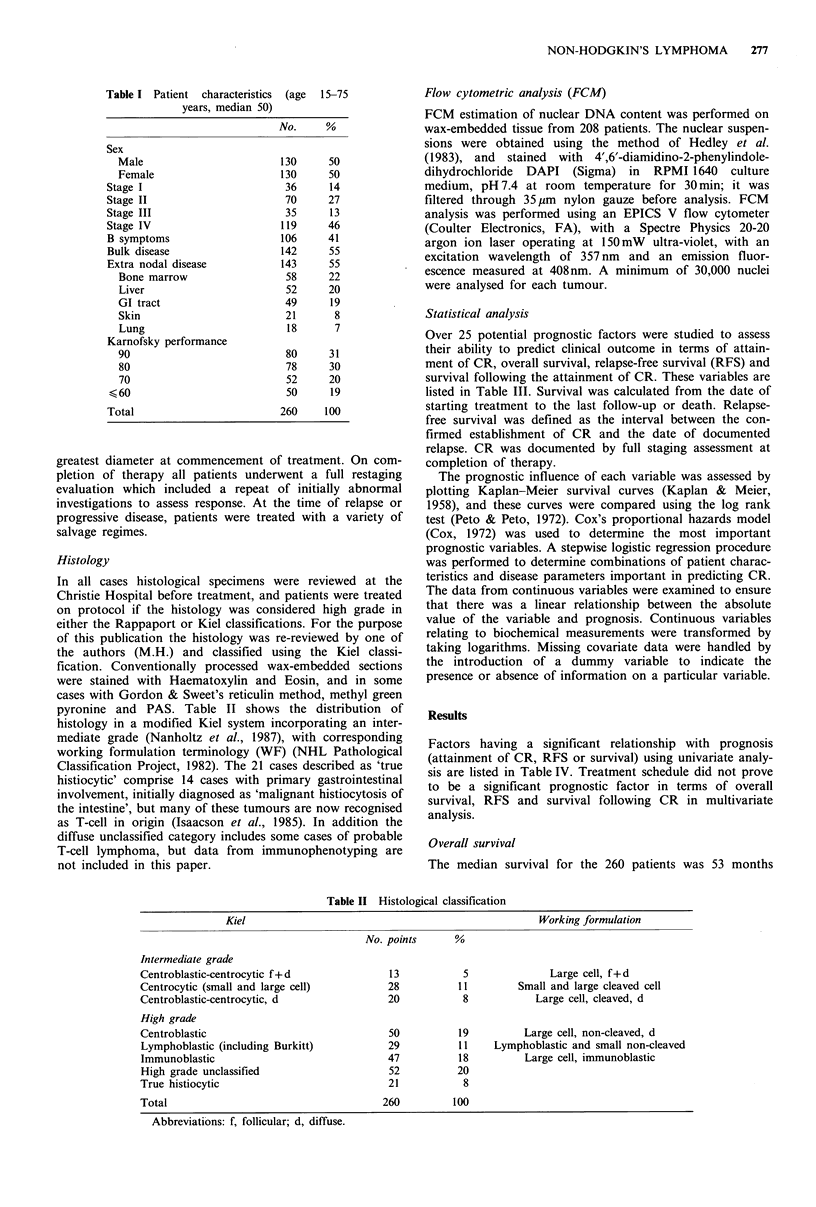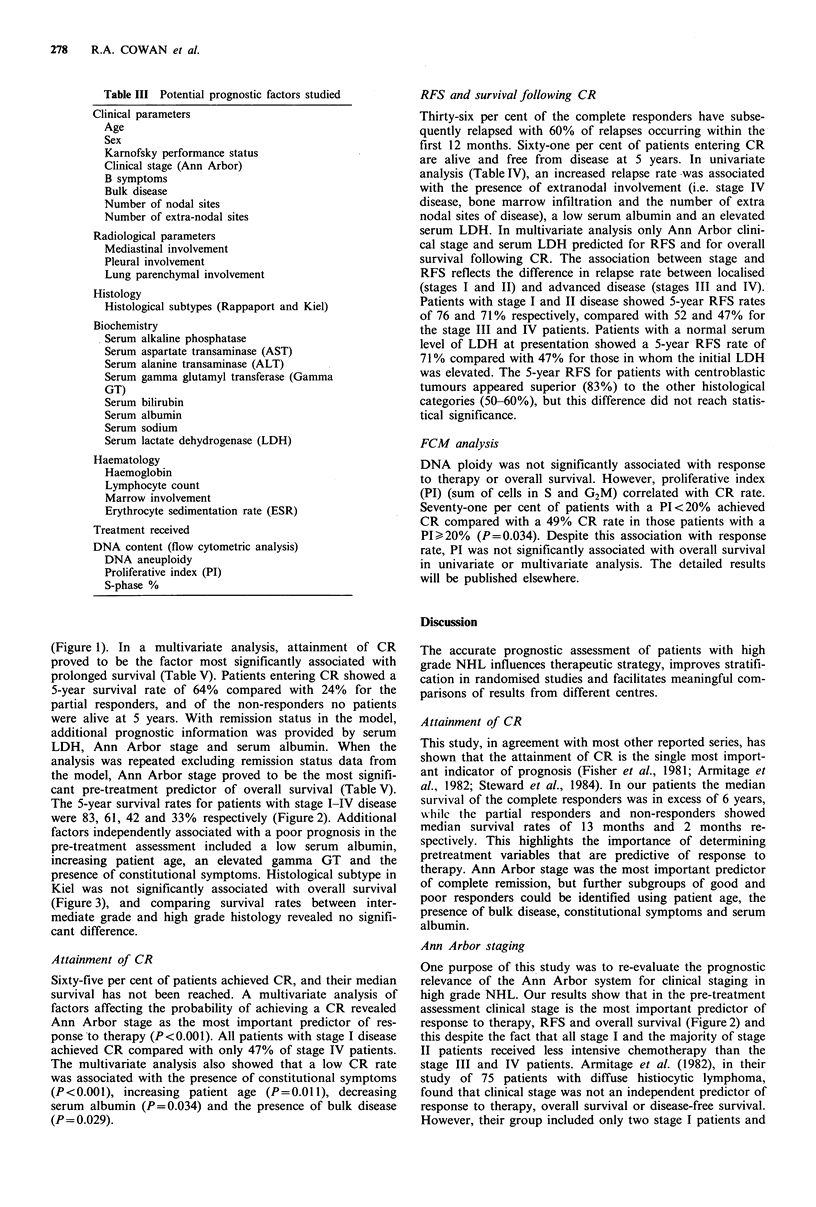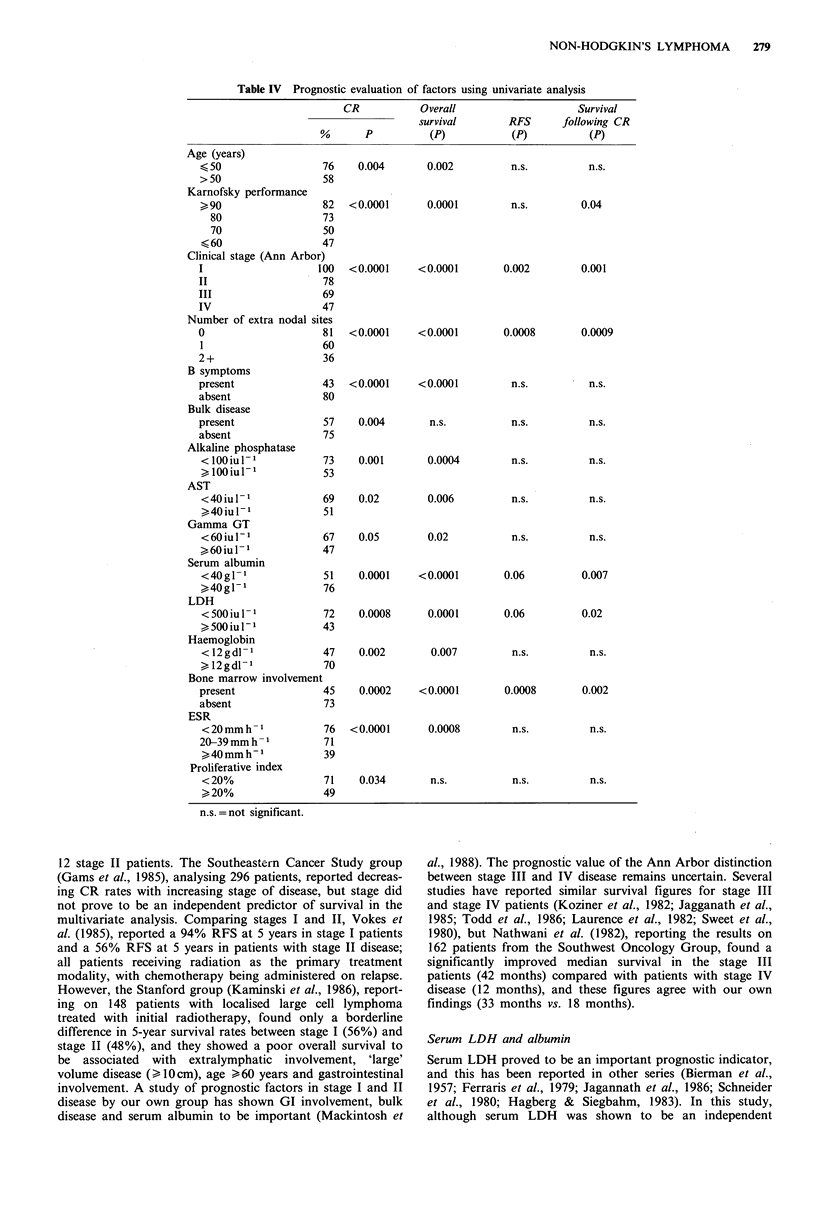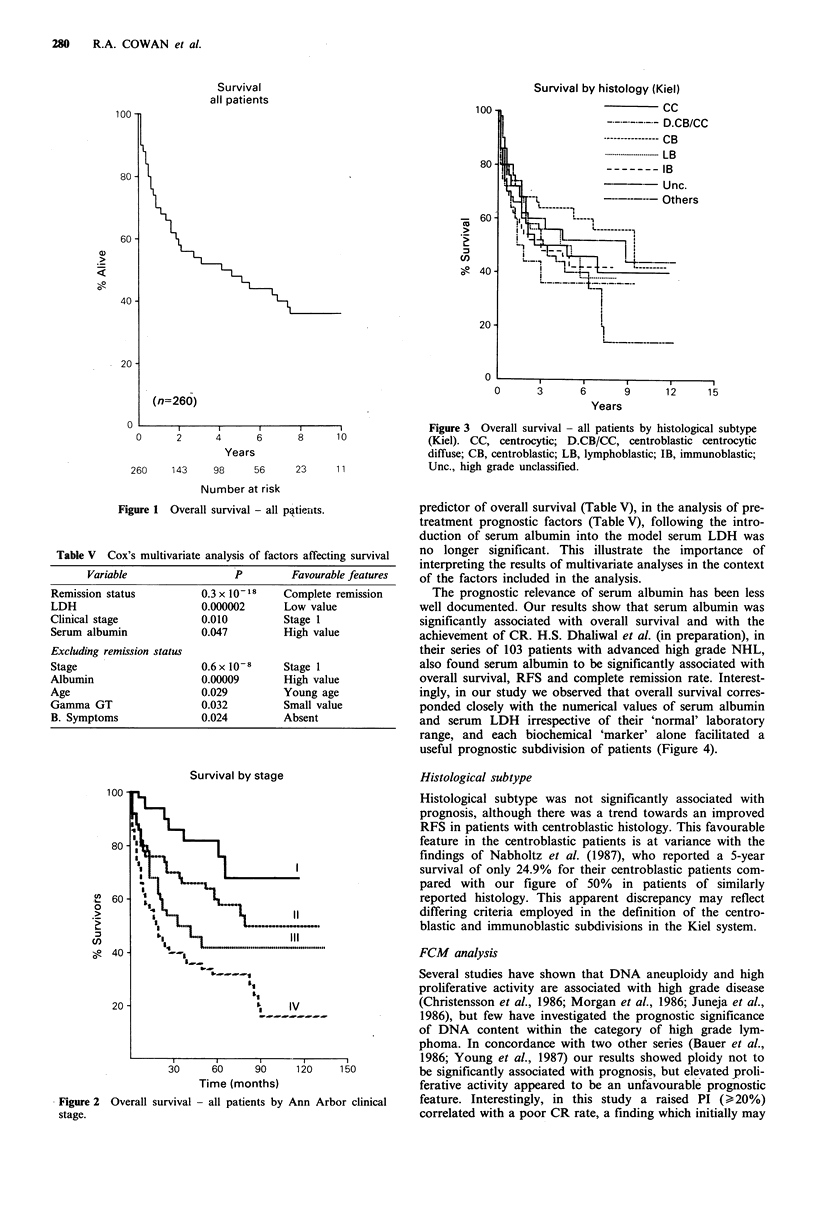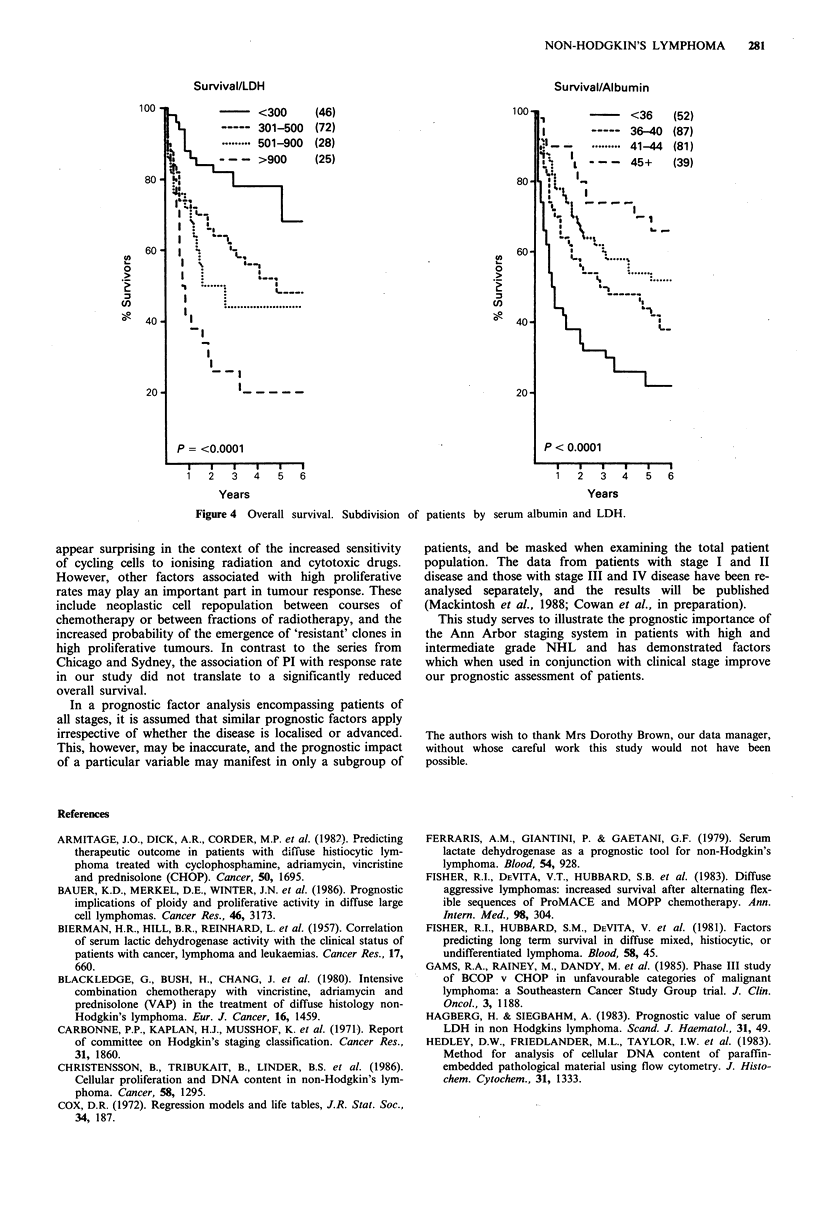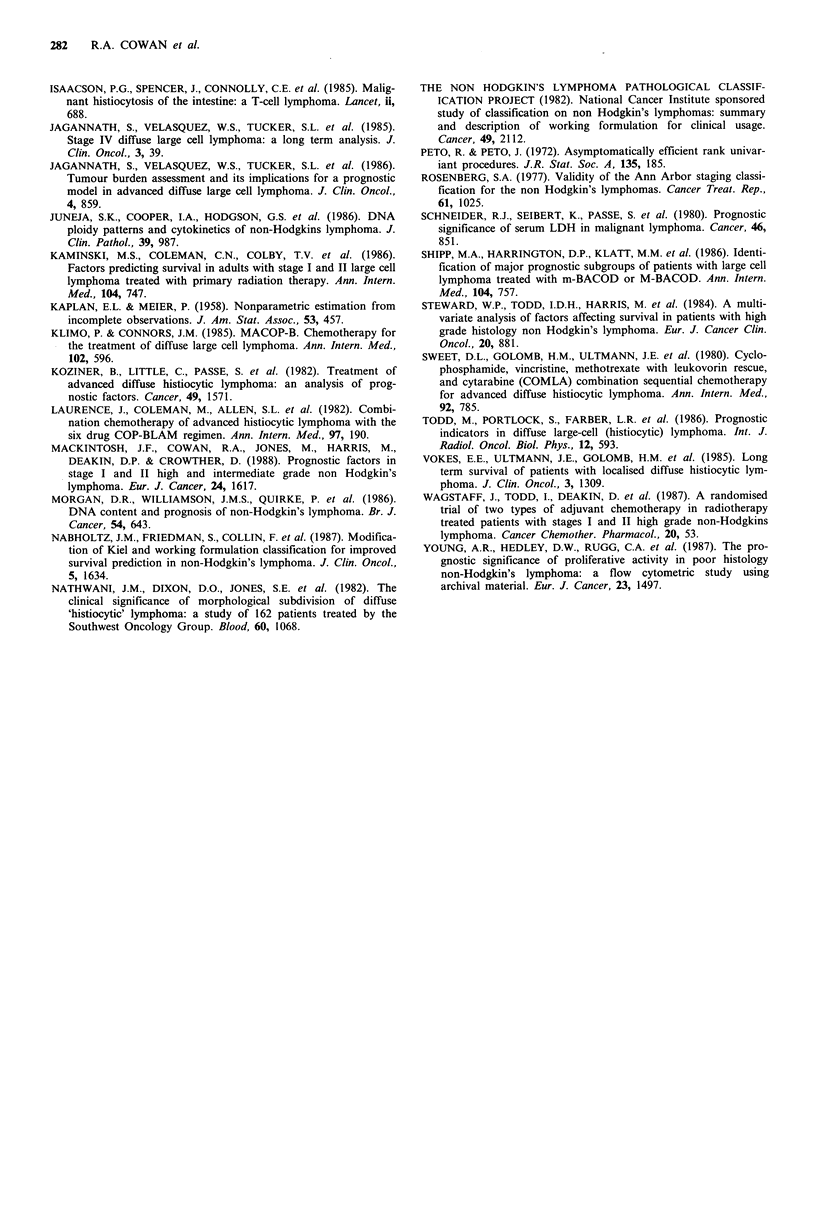# Prognostic factors in high and intermediate grade non-Hodgkin's lymphoma.

**DOI:** 10.1038/bjc.1989.57

**Published:** 1989-02

**Authors:** R. A. Cowan, M. Jones, M. Harris, W. P. Steward, J. A. Radford, J. Wagstaff, D. P. Deakin, D. Crowther

**Affiliations:** CRC Department of Medical Oncology, Christie Hospital and Holt Radium Institute, Manchester, UK.

## Abstract

An analysis of prognostic factors has been performed on 260 patients with high and intermediate grade non-Hodgkin's lymphoma (NHL) treated over an 11-year period between 1975 and 1986. The overall 5-year survival rate was 50% with a median follow-up of 72 months. Over 20 clinical, radiological and laboratory parameters have been studied, including variables reported to be important indicators of prognosis in previous series, and these variables have been subjected to univariate and multivariate analysis. Attainment of complete remission (CR) was the most important predictor of overall survival, low serum lactate dehydrogenase (LDH), limited stage disease and a high serum albumin were also independently associated with prolonged survival in multivariate analysis. After removing remission status from the model, Ann Arbor clinical stage became the most significant pre-treatment prognostic indicator. Sixty-five per cent of patients achieved CR, and a discriminant analysis showed that failure to attain CR was associated with advanced stage disease, constitutional symptoms, increasing patient age, a low serum albumin and the presence of bulk disease. Advanced clinical stage and an elevated serum LDH predicted independently for a poor relapse-free survival, and reduced overall survival following CR. There was no significant correlation between histological subtype in the Kiel classification and prognosis. This study confirms the prognostic significance of remission status and Ann Arbor clinical stage, and illustrates additional factors including serum levels of albumin and LDH, which serve to enhance the pre-treatment prognostic evaluation of patients with unfavourable histology NHL.


Br. J. Cancer (1989), 59, 276–282 © The Macmillan Press Ltd., 1989

### Prognostic factors in high and intermediate grade non-Hodgkin's lymphoma

R.A. Cowan[1], M. Jones[2], M. Harris[3], W.P. Steward[1], J.A. Radford[1], J. Wagstaff[1], D.P. Deakin[4] & D. Crowther[1]

[1]*CRC Department of Medical Oncology;* [2]*Department of Medical Statistics;* [3]*Department of Histopathology and* [4]*Department of Radiotherapy, Christie Hospital and Holt Radium Institute, Wilmslow Road, Manchester M20, UK.*

**Summary** An analysis of prognostic factors has been performed on 260 patients with high and intermediate grade non-Hodgkin's lymphoma (NHL) treated over an 11-year period between 1975 and 1986. The overall 5-year survival rate was 50% with a median follow-up of 72 months. Over 20 clinical, radiological and laboratory parameters have been studied, including variables reported to be important indicators of prognosis in previous series, and these variables have been subjected to univariate and multivariate analysis. Attainment of complete remission (CR) was the most important predictor of overall survival, low serum lactate dehydrogenase (LDH), limited stage disease and a high serum albumin were also independently associated with prolonged survival in multivariate analysis. After removing remission status from the model, Ann Arbor clinical stage became the most significant pre-treatment prognostic indicator. Sixty-five per cent of patients achieved CR, and a discriminant analysis showed that failure to attain CR was associated with advanced stage disease, constitutional symptoms, increasing patient age, a low serum albumin and the presence of bulk disease. Advanced clinical stage and an elevated serum LDH predicted independently for a poor relapse-free survival, and reduced overall survival following CR. There was no significant correlation between histological subtype in the Kiel classification and prognosis. This study confirms the prognostic significance of remission status and Ann Arbor clinical stage, and illustrates additional factors including serum levels of albumin and LDH, which serve to enhance the pre-treatment prognostic evaluation of patients with unfavourable histology NHL.

Over the past decade durable remissions have been achieved in an increasing proportion of patients with high grade NHL and there is evidence that remission rates and survival may be improved by using intensive chemotherapy (Fisher *et al.*, 1983; Klimo & Collins, 1985; Laurence *et al.*, 1982; Blackledge *et al.*, 1980). A more aggressive approach to treatment in this disease is inevitably associated with an increased toxicity and a greater requirement for supportive resources. Clearly an accurate pre-treatment prognostic assessment of patients is required to guide the clinician in the selection of the most appropriate treatment schedule. Currently, in high grade NHL, therapeutic policy is determined predominantly by the extent of disease as defined by the Ann Arbor staging system (Carbonne *et al.*, 1971), and this system has continued to be employed despite our recognition that it harbours some important discrepancies (Rosenberg, 1977). In this study we have set out to reassess the prognostic relevance of the Ann Arbor system in high grade NHL and, further, have attempted to identify additional patient characteristics and disease parameters which may be used in conjunction with the Ann Arbor system to improve our prognostic evaluation of patients.

Retrospective studies performed over the past 10 years have reported a number of factors having influence on prognosis in high grade NHL. However, the interpretation of data from many series has been hampered by small patient numbers and the fact that only few centres have employed multivariate analyses (Jagganath *et al.*, 1985; Armitage *et al.*, 1982; Steward *et al.*, 1984; Todd *et al.*, 1986; Shipp *et al.*, 1986; Fisher *et al.*, 1981; Koziner *et al.*, 1982). Comparison of results from different studies is further compromised by the lack of uniformity in the variables analysed, and in many publications the potential prognostic factors investigated are not clearly listed.

In an attempt to re-examine the prognostic evaluation of patients, we have collected data, including all variables previously reported as having significant prognostic importance in high grade NHL, and subjected these data to multivariate analysis.

Correspondence: R.A. Cowan.
Received 21 July 1988, and in revised form, 25 October 1988.

### Materials and methods

### *Patient details*

The Manchester Lymphoma Group entered 260 previously untreated patients in treatment protocols for high grade NHL between 1975 and 1986. The patient selection criteria included: age 15–75 years inclusive, no previous treatment for NHL, no previous history of malignancy and no accompanying medical condition which would preclude either chemotherapy or radiation treatment. The patient details are shown in Table I. Two hundred and six patients presenting with Ann Arbor stage II (with bulk nodal involvement), stage III and IV disease received a 6-week course of induction chemotherapy with VAP (vincristine, adriamycin and prednisolone) (Blackledge *et al.*, 1980). The remaining patients with stage I and II (non-bulky) disease received initial radiotherapy and were subsequently randomised to receive either adjuvant VAP or 3-weekly cycles of CMOPP (cyclophosphamide, mustine, vincristine, procarbazine and prednisolone) (Wagstaff *et al.*, 1987). Involved field radiotherapy was given to the original site of disease in patients with stage I and stage II lymphoma to a dose of 3,000 cGy over 3 weeks. Patients with stage III and IV disease were given radiotherapy to previously involved bulky sites following induction chemotherapy. Those patients in CR received 2,500 cGy fractionated over 8 days, whereas patients with residual tumour following induction chemotherapy received 3,000–3,500 cGy with wide margins over 3 weeks. The median follow-up for the group is 72 months.

All patients were staged using the Ann Arbor system. Pre-treatment evaluation included a detailed history and physical examination, a full blood count and ESR, biochemical profile (including liver enzymes and LDH), a bone marrow aspirate and trephine from a single site, CSF examination, chest radiograph and computed tomographic scan (CT) of the abdomen and pelvis. Laparotomies were not performed for routine staging purposes, and percutaneous liver biopsy was undertaken in only a minority of cases. Isotopic bone scans, abdominal ultrasound scans and barium GI tract studies were performed when clinically indicated. Bulk disease was defined as tumour measuring more than 5 cm in

**Table I** Patient characteristics (age 15–75 years, median 50)

| | *No.* | *%* |
|---|---|---|
| Sex | | |
| Male | 130 | 50 |
| Female | 130 | 50 |
| Stage I | 36 | 14 |
| Stage II | 70 | 27 |
| Stage III | 35 | 13 |
| Stage IV | 119 | 46 |
| B symptoms | 106 | 41 |
| Bulk disease | 142 | 55 |
| Extra nodal disease | 143 | 55 |
| Bone marrow | 58 | 22 |
| Liver | 52 | 20 |
| GI tract | 49 | 19 |
| Skin | 21 | 8 |
| Lung | 18 | 7 |
| Karnofsky performance | | |
| 90 | 80 | 31 |
| 80 | 78 | 30 |
| 70 | 52 | 20 |
| ⩽60 | 50 | 19 |
| Total | 260 | 100 |

greatest diameter at commencement of treatment. On completion of therapy all patients underwent a full restaging evaluation which included a repeat of initially abnormal investigations to assess response. At the time of relapse or progressive disease, patients were treated with a variety of salvage regimes.

*Histology*

In all cases histological specimens were reviewed at the Christie Hospital before treatment, and patients were treated on protocol if the histology was considered high grade in either the Rappaport or Kiel classifications. For the purpose of this publication the histology was re-reviewed by one of the authors (M.H.) and classified using the Kiel classification. Conventionally processed wax-embedded sections were stained with Haematoxylin and Eosin, and in some cases with Gordon & Sweet's reticulin method, methyl green pyronine and PAS. Table II shows the distribution of histology in a modified Kiel system incorporating an intermediate grade (Nanholtz *et al.*, 1987), with corresponding working formulation terminology (WF) (NHL Pathological Classification Project, 1982). The 21 cases described as 'true histiocytic' comprise 14 cases with primary gastrointestinal involvement, initially diagnosed as 'malignant histiocytosis of the intestine', but many of these tumours are now recognised as T-cell in origin (Isaacson *et al.*, 1985). In addition the diffuse unclassified category includes some cases of probable T-cell lymphoma, but data from immunophenotyping are not included in this paper.

*Flow cytometric analysis (FCM)*

FCM estimation of nuclear DNA content was performed on wax-embedded tissue from 208 patients. The nuclear suspensions were obtained using the method of Hedley *et al.* (1983), and stained with 4′,6′-diamidino-2-phenylindole-dihydrochloride DAPI (Sigma) in RPMI 1640 culture medium, pH 7.4 at room temperature for 30 min; it was filtered through 35 $\mu$m nylon gauze before analysis. FCM analysis was performed using an EPICS V flow cytometer (Coulter Electronics, FA), with a Spectre Physics 20-20 argon ion laser operating at 150 mW ultra-violet, with an excitation wavelength of 357 nm and an emission fluorescence measured at 408 nm. A minimum of 30,000 nuclei were analysed for each tumour.

*Statistical analysis*

Over 25 potential prognostic factors were studied to assess their ability to predict clinical outcome in terms of attainment of CR, overall survival, relapse-free survival (RFS) and survival following the attainment of CR. These variables are listed in Table III. Survival was calculated from the date of starting treatment to the last follow-up or death. Relapse-free survival was defined as the interval between the confirmed establishment of CR and the date of documented relapse. CR was documented by full staging assessment at completion of therapy.

The prognostic influence of each variable was assessed by plotting Kaplan–Meier survival curves (Kaplan & Meier, 1958), and these curves were compared using the log rank test (Peto & Peto, 1972). Cox's proportional hazards model (Cox, 1972) was used to determine the most important prognostic variables. A stepwise logistic regression procedure was performed to determine combinations of patient characteristics and disease parameters important in predicting CR. The data from continuous variables were examined to ensure that there was a linear relationship between the absolute value of the variable and prognosis. Continuous variables relating to biochemical measurements were transformed by taking logarithms. Missing covariate data were handled by the introduction of a dummy variable to indicate the presence or absence of information on a particular variable.

### Results

Factors having a significant relationship with prognosis (attainment of CR, RFS or survival) using univariate analysis are listed in Table IV. Treatment schedule did not prove to be a significant prognostic factor in terms of overall survival, RFS and survival following CR in multivariate analysis.

*Overall survival*

The median survival for the 260 patients was 53 months

**Table II** Histological classification

| *Kiel* | *No. points* | *%* | *Working formulation* |
|---|---|---|---|
| *Intermediate grade* | | | |
| Centroblastic-centrocytic f+d | 13 | 5 | Large cell, f+d |
| Centrocytic (small and large cell) | 28 | 11 | Small and large cleaved cell |
| Centroblastic-centrocytic, d | 20 | 8 | Large cell, cleaved, d |
| *High grade* | | | |
| Centroblastic | 50 | 19 | Large cell, non-cleaved, d |
| Lymphoblastic (including Burkitt) | 29 | 11 | Lymphoblastic and small non-cleaved |
| Immunoblastic | 47 | 18 | Large cell, immunoblastic |
| High grade unclassified | 52 | 20 | |
| True histiocytic | 21 | 8 | |
| Total | 260 | 100 | |

Abbreviations: f, follicular; d, diffuse.

**Table III** Potential prognostic factors studied

| |
|---|
| Clinical parameters |
| Age |
| Sex |
| Karnofsky performance status |
| Clinical stage (Ann Arbor) |
| B symptoms |
| Bulk disease |
| Number of nodal sites |
| Number of extra-nodal sites |
| Radiological parameters |
| Mediastinal involvement |
| Pleural involvement |
| Lung parenchymal involvement |
| Histology |
| Histological subtypes (Rappaport and Kiel) |
| Biochemistry |
| Serum alkaline phosphatase |
| Serum aspartate transaminase (AST) |
| Serum alanine transaminase (ALT) |
| Serum gamma glutamyl transferase (Gamma GT) |
| Serum bilirubin |
| Serum albumin |
| Serum sodium |
| Serum lactate dehydrogenase (LDH) |
| Haematology |
| Haemoglobin |
| Lymphocyte count |
| Marrow involvement |
| Erythrocyte sedimentation rate (ESR) |
| Treatment received |
| DNA content (flow cytometric analysis) |
| DNA aneuploidy |
| Proliferative index (PI) |
| S-phase % |

(Figure 1). In a multivariate analysis, attainment of CR proved to be the factor most significantly associated with prolonged survival (Table V). Patients entering CR showed a 5-year survival rate of 64% compared with 24% for the partial responders, and of the non-responders no patients were alive at 5 years. With remission status in the model, additional prognostic information was provided by serum LDH, Ann Arbor stage and serum albumin. When the analysis was repeated excluding remission status data from the model, Ann Arbor stage proved to be the most significant pre-treatment predictor of overall survival (Table V). The 5-year survival rates for patients with stage I–IV disease were 83, 61, 42 and 33% respectively (Figure 2). Additional factors independently associated with a poor prognosis in the pre-treatment assessment included a low serum albumin, increasing patient age, an elevated gamma GT and the presence of constitutional symptoms. Histological subtype in Kiel was not significantly associated with overall survival (Figure 3), and comparing survival rates between intermediate grade and high grade histology revealed no significant difference.

*Attainment of CR*

Sixty-five per cent of patients achieved CR, and their median survival has not been reached. A multivariate analysis of factors affecting the probability of achieving a CR revealed Ann Arbor stage as the most important predictor of response to therapy ($P<0.001$). All patients with stage I disease achieved CR compared with only 47% of stage IV patients. The multivariate analysis also showed that a low CR rate was associated with the presence of constitutional symptoms ($P<0.001$), increasing patient age ($P=0.011$), decreasing serum albumin ($P=0.034$) and the presence of bulk disease ($P=0.029$).

*RFS and survival following CR*

Thirty-six per cent of the complete responders have subsequently relapsed with 60% of relapses occurring within the first 12 months. Sixty-one per cent of patients entering CR are alive and free from disease at 5 years. In univariate analysis (Table IV), an increased relapse rate was associated with the presence of extranodal involvement (i.e. stage IV disease, bone marrow infiltration and the number of extra nodal sites of disease), a low serum albumin and an elevated serum LDH. In multivariate analysis only Ann Arbor clinical stage and serum LDH predicted for RFS and for overall survival following CR. The association between stage and RFS reflects the difference in relapse rate between localised (stages I and II) and advanced disease (stages III and IV). Patients with stage I and II disease showed 5-year RFS rates of 76 and 71% respectively, compared with 52 and 47% for the stage III and IV patients. Patients with a normal serum level of LDH at presentation showed a 5-year RFS rate of 71% compared with 47% for those in whom the initial LDH was elevated. The 5-year RFS for patients with centroblastic tumours appeared superior (83%) to the other histological categories (50–60%), but this difference did not reach statistical significance.

*FCM analysis*

DNA ploidy was not significantly associated with response to therapy or overall survival. However, proliferative index (PI) (sum of cells in S and $G_2M$) correlated with CR rate. Seventy-one per cent of patients with a PI$<$20% achieved CR compared with a 49% CR rate in those patients with a PI$\geqslant$20% ($P=0.034$). Despite this association with response rate, PI was not significantly associated with overall survival in univariate or multivariate analysis. The detailed results will be published elsewhere.

### Discussion

The accurate prognostic assessment of patients with high grade NHL influences therapeutic strategy, improves stratification in randomised studies and facilitates meaningful comparisons of results from different centres.

*Attainment of CR*

This study, in agreement with most other reported series, has shown that the attainment of CR is the single most important indicator of prognosis (Fisher *et al.*, 1981; Armitage *et al.*, 1982; Steward *et al.*, 1984). In our patients the median survival of the complete responders was in excess of 6 years, while the partial responders and non-responders showed median survival rates of 13 months and 2 months respectively. This highlights the importance of determining pretreatment variables that are predictive of response to therapy. Ann Arbor stage was the most important predictor of complete remission, but further subgroups of good and poor responders could be identified using patient age, the presence of bulk disease, constitutional symptoms and serum albumin.

*Ann Arbor staging*

One purpose of this study was to re-evaluate the prognostic relevance of the Ann Arbor system for clinical staging in high grade NHL. Our results show that in the pre-treatment assessment clinical stage is the most important predictor of response to therapy, RFS and overall survival (Figure 2) and this despite the fact that all stage I and the majority of stage II patients received less intensive chemotherapy than the stage III and IV patients. Armitage *et al.* (1982), in their study of 75 patients with diffuse histiocytic lymphoma, found that clinical stage was not an independent predictor of response to therapy, overall survival or disease-free survival. However, their group included only two stage I patients and

**Table IV** Prognostic evaluation of factors using univariate analysis

| | *CR* % | *CR* P | *Overall survival (P)* | *RFS (P)* | *Survival following CR (P)* |
|---|---|---|---|---|---|
| Age (years) | | | | | |
| ⩽50 | 76 | 0.004 | 0.002 | n.s. | n.s. |
| >50 | 58 | | | | |
| Karnofsky performance | | | | | |
| ⩾90 | 82 | <0.0001 | 0.0001 | n.s. | 0.04 |
| 80 | 73 | | | | |
| 70 | 50 | | | | |
| ⩽60 | 47 | | | | |
| Clinical stage (Ann Arbor) | | | | | |
| I | 100 | <0.0001 | <0.0001 | 0.002 | 0.001 |
| II | 78 | | | | |
| III | 69 | | | | |
| IV | 47 | | | | |
| Number of extra nodal sites | | | | | |
| 0 | 81 | <0.0001 | <0.0001 | 0.0008 | 0.0009 |
| 1 | 60 | | | | |
| 2+ | 36 | | | | |
| B symptoms | | | | | |
| present | 43 | <0.0001 | <0.0001 | n.s. | n.s. |
| absent | 80 | | | | |
| Bulk disease | | | | | |
| present | 57 | 0.004 | n.s. | n.s. | n.s. |
| absent | 75 | | | | |
| Alkaline phosphatase | | | | | |
| <100 iu l$^{-1}$ | 73 | 0.001 | 0.0004 | n.s. | n.s. |
| ⩾100 iu l$^{-1}$ | 53 | | | | |
| AST | | | | | |
| <40 iu l$^{-1}$ | 69 | 0.02 | 0.006 | n.s. | n.s. |
| ⩾40 iu l$^{-1}$ | 51 | | | | |
| Gamma GT | | | | | |
| <60 iu l$^{-1}$ | 67 | 0.05 | 0.02 | n.s. | n.s. |
| ⩾60 iu l$^{-1}$ | 47 | | | | |
| Serum albumin | | | | | |
| <40 g l$^{-1}$ | 51 | 0.0001 | <0.0001 | 0.06 | 0.007 |
| ⩾40 g l$^{-1}$ | 76 | | | | |
| LDH | | | | | |
| <500 iu l$^{-1}$ | 72 | 0.0008 | 0.0001 | 0.06 | 0.02 |
| ⩾500 iu l$^{-1}$ | 43 | | | | |
| Haemoglobin | | | | | |
| <12 g dl$^{-1}$ | 47 | 0.002 | 0.007 | n.s. | n.s. |
| ⩾12 g dl$^{-1}$ | 70 | | | | |
| Bone marrow involvement | | | | | |
| present | 45 | 0.0002 | <0.0001 | 0.0008 | 0.002 |
| absent | 73 | | | | |
| ESR | | | | | |
| <20 mm h$^{-1}$ | 76 | <0.0001 | 0.0008 | n.s. | n.s. |
| 20–39 mm h$^{-1}$ | 71 | | | | |
| ⩾40 mm h$^{-1}$ | 39 | | | | |
| Proliferative index | | | | | |
| <20% | 71 | 0.034 | n.s. | n.s. | n.s. |
| ⩾20% | 49 | | | | |

n.s. = not significant.

12 stage II patients. The Southeastern Cancer Study group (Gams *et al.*, 1985), analysing 296 patients, reported decreasing CR rates with increasing stage of disease, but stage did not prove to be an independent predictor of survival in the multivariate analysis. Comparing stages I and II, Vokes *et al.* (1985), reported a 94% RFS at 5 years in stage I patients and a 56% RFS at 5 years in patients with stage II disease; all patients receiving radiation as the primary treatment modality, with chemotherapy being administered on relapse. However, the Stanford group (Kaminski *et al.*, 1986), reporting on 148 patients with localised large cell lymphoma treated with initial radiotherapy, found only a borderline difference in 5-year survival rates between stage I (56%) and stage II (48%), and they showed a poor overall survival to be associated with extralymphatic involvement, 'large' volume disease (⩾10 cm), age ⩾60 years and gastrointestinal involvement. A study of prognostic factors in stage I and II disease by our own group has shown GI involvement, bulk disease and serum albumin to be important (Mackintosh *et al.*, 1988). The prognostic value of the Ann Arbor distinction between stage III and IV disease remains uncertain. Several studies have reported similar survival figures for stage III and stage IV patients (Koziner *et al.*, 1982; Jagganath *et al.*, 1985; Todd *et al.*, 1986; Laurence *et al.*, 1982; Sweet *et al.*, 1980), but Nathwani *et al.* (1982), reporting the results on 162 patients from the Southwest Oncology Group, found a significantly improved median survival in the stage III patients (42 months) compared with patients with stage IV disease (12 months), and these figures agree with our own findings (33 months *vs.* 18 months).

### *Serum LDH and albumin*

Serum LDH proved to be an important prognostic indicator, and this has been reported in other series (Bierman *et al.*, 1957; Ferraris *et al.*, 1979; Jagannath *et al.*, 1986; Schneider *et al.*, 1980; Hagberg & Siegbahm, 1983). In this study, although serum LDH was shown to be an independent

**Figure 1** Overall survival – all patients.

**Table V** Cox's multivariate analysis of factors affecting survival

| *Variable* | *P* | *Favourable features* |
|---|---|---|
| Remission status | $0.3 \times 10^{-18}$ | Complete remission |
| LDH | 0.000002 | Low value |
| Clinical stage | 0.010 | Stage 1 |
| Serum albumin | 0.047 | High value |
| *Excluding remission status* | | |
| Stage | $0.6 \times 10^{-8}$ | Stage 1 |
| Albumin | 0.00009 | High value |
| Age | 0.029 | Young age |
| Gamma GT | 0.032 | Small value |
| B. Symptoms | 0.024 | Absent |

**Figure 2** Overall survival – all patients by Ann Arbor clinical stage.

**Figure 3** Overall survival – all patients by histological subtype (Kiel). CC, centrocytic; D.CB/CC, centroblastic centrocytic diffuse; CB, centroblastic; LB, lymphoblastic; IB, immunoblastic; Unc., high grade unclassified.

predictor of overall survival (Table V), in the analysis of pre-treatment prognostic factors (Table V), following the introduction of serum albumin into the model serum LDH was no longer significant. This illustrate the importance of interpreting the results of multivariate analyses in the context of the factors included in the analysis.

The prognostic relevance of serum albumin has been less well documented. Our results show that serum albumin was significantly associated with overall survival and with the achievement of CR. H.S. Dhaliwal *et al.* (in preparation), in their series of 103 patients with advanced high grade NHL, also found serum albumin to be significantly associated with overall survival, RFS and complete remission rate. Interestingly, in our study we observed that overall survival corresponded closely with the numerical values of serum albumin and serum LDH irrespective of their 'normal' laboratory range, and each biochemical 'marker' alone facilitated a useful prognostic subdivision of patients (Figure 4).

### *Histological subtype*

Histological subtype was not significantly associated with prognosis, although there was a trend towards an improved RFS in patients with centroblastic histology. This favourable feature in the centroblastic patients is at variance with the findings of Nabholtz *et al.* (1987), who reported a 5-year survival of only 24.9% for their centroblastic patients compared with our figure of 50% in patients of similarly reported histology. This apparent discrepancy may reflect differing criteria employed in the definition of the centroblastic and immunoblastic subdivisions in the Kiel system.

### *FCM analysis*

Several studies have shown that DNA aneuploidy and high proliferative activity are associated with high grade disease (Christensson *et al.*, 1986; Morgan *et al.*, 1986; Juneja *et al.*, 1986), but few have investigated the prognostic significance of DNA content within the category of high grade lymphoma. In concordance with two other series (Bauer *et al.*, 1986; Young *et al.*, 1987) our results showed ploidy not to be significantly associated with prognosis, but elevated proliferative activity appeared to be an unfavourable prognostic feature. Interestingly, in this study a raised PI (⩾20%) correlated with a poor CR rate, a finding which initially may

**Figure 4** Overall survival. Subdivision of patients by serum albumin and LDH.

appear surprising in the context of the increased sensitivity of cycling cells to ionising radiation and cytotoxic drugs. However, other factors associated with high proliferative rates may play an important part in tumour response. These include neoplastic cell repopulation between courses of chemotherapy or between fractions of radiotherapy, and the increased probability of the emergence of 'resistant' clones in high proliferative tumours. In contrast to the series from Chicago and Sydney, the association of PI with response rate in our study did not translate to a significantly reduced overall survival.

In a prognostic factor analysis encompassing patients of all stages, it is assumed that similar prognostic factors apply irrespective of whether the disease is localised or advanced. This, however, may be inaccurate, and the prognostic impact of a particular variable may manifest in only a subgroup of patients, and be masked when examining the total patient population. The data from patients with stage I and II disease and those with stage III and IV disease have been re-analysed separately, and the results will be published (Mackintosh *et al.*, 1988; Cowan *et al.*, in preparation).

This study serves to illustrate the prognostic importance of the Ann Arbor staging system in patients with high and intermediate grade NHL and has demonstrated factors which when used in conjunction with clinical stage improve our prognostic assessment of patients.

The authors wish to thank Mrs Dorothy Brown, our data manager, without whose careful work this study would not have been possible.

### References

ARMITAGE, J.O., DICK, A.R., CORDER, M.P. *et al.* (1982). Predicting therapeutic outcome in patients with diffuse histiocytic lymphoma treated with cyclophosphamine, adriamycin, vincristine and prednisolone (CHOP). *Cancer*, **50**, 1695.

BAUER, K.D., MERKEL, D.E., WINTER, J.N. *et al.* (1986). Prognostic implications of ploidy and proliferative activity in diffuse large cell lymphomas. *Cancer Res.*, **46**, 3173.

BIERMAN, H.R., HILL, B.R., REINHARD, L. *et al.* (1957). Correlation of serum lactic dehydrogenase activity with the clinical status of patients with cancer, lymphoma and leukaemias. *Cancer Res.*, **17**, 660.

BLACKLEDGE, G., BUSH, H., CHANG, J. *et al.* (1980). Intensive combination chemotherapy with vincristine, adriamycin and prednisolone (VAP) in the treatment of diffuse histology non-Hodgkin's lymphoma. *Eur. J. Cancer*, **16**, 1459.

CARBONNE, P.P., KAPLAN, H.J., MUSSHOF, K. *et al.* (1971). Report of committee on Hodgkin's staging classification. *Cancer Res.*, **31**, 1860.

CHRISTENSSON, B., TRIBUKAIT, B., LINDER, B.S. *et al.* (1986). Cellular proliferation and DNA content in non-Hodgkin's lymphoma. *Cancer*, **58**, 1295.

COX, D.R. (1972). Regression models and life tables, *J.R. Stat. Soc.*, **34**, 187.

FERRARIS, A.M., GIANTINI, P. & GAETANI, G.F. (1979). Serum lactate dehydrogenase as a prognostic tool for non-Hodgkin's lymphoma. *Blood*, **54**, 928.

FISHER, R.I., DEVITA, V.T., HUBBARD, S.B. *et al.* (1983). Diffuse aggressive lymphomas: increased survival after alternating flexible sequences of ProMACE and MOPP chemotherapy. *Ann. Intern. Med.*, **98**, 304.

FISHER, R.I., HUBBARD, S.M., DEVITA, V. *et al.* (1981). Factors predicting long term survival in diffuse mixed, histiocytic, or undifferentiated lymphoma. *Blood*, **58**, 45.

GAMS, R.A., RAINEY, M., DANDY, M. *et al.* (1985). Phase III study of BCOP v CHOP in unfavourable categories of malignant lymphoma: a Southeastern Cancer Study Group trial. *J. Clin. Oncol.*, **3**, 1188.

HAGBERG, H. & SIEGBAHM, A. (1983). Prognostic value of serum LDH in non Hodgkins lymphoma. *Scand. J. Haematol.*, **31**, 49.

HEDLEY, D.W., FRIEDLANDER, M.L., TAYLOR, I.W. *et al.* (1983). Method for analysis of cellular DNA content of paraffin-embedded pathological material using flow cytometry. *J. Histochem. Cytochem.*, **31**, 1333.

ISAACSON, P.G., SPENCER, J., CONNOLLY, C.E. *et al.* (1985). Malignant histiocytosis of the intestine: a T-cell lymphoma. *Lancet*, **ii**, 688.

JAGANNATH, S., VELASQUEZ, W.S., TUCKER, S.L. *et al.* (1985). Stage IV diffuse large cell lymphoma: a long term analysis. *J. Clin. Oncol.*, **3**, 39.

JAGANNATH, S., VELASQUEZ, W.S., TUCKER, S.L. *et al.* (1986). Tumour burden assessment and its implications for a prognostic model in advanced diffuse large cell lymphoma. *J. Clin. Oncol.*, **4**, 859.

JUNEJA, S.K., COOPER, I.A., HODGSON, G.S. *et al.* (1986). DNA ploidy patterns and cytokinetics of non-Hodgkins lymphoma. *J. Clin. Pathol.*, **39**, 987.

KAMINSKI, M.S., COLEMAN, C.N., COLBY, T.V. *et al.* (1986). Factors predicting survival in adults with stage I and II large cell lymphoma treated with primary radiation therapy. *Ann. Intern. Med.*, **104**, 747.

KAPLAN, E.L. & MEIER, P. (1958). Nonparametric estimation from incomplete observations. *J. Am. Stat. Assoc.*, **53**, 457.

KLIMO, P. & CONNORS, J.M. (1985). MACOP-B. Chemotherapy for the treatment of diffuse large cell lymphoma. *Ann. Intern. Med.*, **102**, 596.

KOZINER, B., LITTLE, C., PASSE, S. *et al.* (1982). Treatment of advanced diffuse histiocytic lymphoma: an analysis of prognostic factors. *Cancer*, **49**, 1571.

LAURENCE, J., COLEMAN, M., ALLEN, S.L. *et al.* (1982). Combination chemotherapy of advanced histiocytic lymphoma with the six drug COP-BLAM regimen. *Ann. Intern. Med.*, **97**, 190.

MACKINTOSH, J.F., COWAN, R.A., JONES, M., HARRIS, M., DEAKIN, D.P. & CROWTHER, D. (1988). Prognostic factors in stage I and II high and intermediate grade non Hodgkin's lymphoma. *Eur. J. Cancer*, **24**, 1617.

MORGAN, D.R., WILLIAMSON, J.M.S., QUIRKE, P. *et al.* (1986). DNA content and prognosis of non-Hodgkin's lymphoma. *Br. J. Cancer*, **54**, 643.

NABHOLTZ, J.M., FRIEDMAN, S., COLLIN, F. *et al.* (1987). Modification of Kiel and working formulation classification for improved survival prediction in non-Hodgkin's lymphoma. *J. Clin. Oncol.*, **5**, 1634.

NATHWANI, J.M., DIXON, D.O., JONES, S.E. *et al.* (1982). The clinical significance of morphological subdivision of diffuse 'histiocytic' lymphoma: a study of 162 patients treated by the Southwest Oncology Group. *Blood*, **60**, 1068.

THE NON HODGKIN'S LYMPHOMA PATHOLOGICAL CLASSIFICATION PROJECT (1982). National Cancer Institute sponsored study of classification on non Hodgkin's lymphomas: summary and description of working formulation for clinical usage. *Cancer*, **49**, 2112.

PETO, R. & PETO, J. (1972). Asymptomatically efficient rank univariant procedures. *J.R. Stat. Soc. A*, **135**, 185.

ROSENBERG, S.A. (1977). Validity of the Ann Arbor staging classification for the non Hodgkin's lymphomas. *Cancer Treat. Rep.*, **61**, 1025.

SCHNEIDER, R.J., SEIBERT, K., PASSE, S. *et al.* (1980). Prognostic significance of serum LDH in malignant lymphoma. *Cancer*, **46**, 851.

SHIPP, M.A., HARRINGTON, D.P., KLATT, M.M. *et al.* (1986). Identification of major prognostic subgroups of patients with large cell lymphoma treated with m-BACOD or M-BACOD. *Ann. Intern. Med.*, **104**, 757.

STEWARD, W.P., TODD, I.D.H., HARRIS, M. *et al.* (1984). A multivariate analysis of factors affecting survival in patients with high grade histology non Hodgkin's lymphoma. *Eur. J. Cancer Clin. Oncol.*, **20**, 881.

SWEET, D.L., GOLOMB, H.M., ULTMANN, J.E. *et al.* (1980). Cyclophosphamide, vincristine, methotrexate with leukovorin rescue, and cytarabine (COMLA) combination sequential chemotherapy for advanced diffuse histiocytic lymphoma. *Ann. Intern. Med.*, **92**, 785.

TODD, M., PORTLOCK, S., FARBER, L.R. *et al.* (1986). Prognostic indicators in diffuse large-cell (histiocytic) lymphoma. *Int. J. Radiol. Oncol. Biol. Phys.*, **12**, 593.

VOKES, E.E., ULTMANN, J.E., GOLOMB, H.M. *et al.* (1985). Long term survival of patients with localised diffuse histiocytic lymphoma. *J. Clin. Oncol.*, **3**, 1309.

WAGSTAFF, J., TODD, I., DEAKIN, D. *et al.* (1987). A randomised trial of two types of adjuvant chemotherapy in radiotherapy treated patients with stages I and II high grade non-Hodgkins lymphoma. *Cancer Chemother. Pharmacol.*, **20**, 53.

YOUNG, A.R., HEDLEY, D.W., RUGG, C.A. *et al.* (1987). The prognostic significance of proliferative activity in poor histology non-Hodgkin's lymphoma: a flow cytometric study using archival material. *Eur. J. Cancer*, **23**, 1497.